# External Quality Assessment of Sputum Smear Microscopy in Tuberculosis Laboratories in Sughd, Tajikistan

**DOI:** 10.5195/cajgh.2015.230

**Published:** 2016-03-04

**Authors:** Eva Chang, Fabio Luelmo, Zamira Baydulloeva, Marija Joncevska, Guljamol Kasymova, Oktam Bobokhojaev, Tom Mohr

**Affiliations:** 1 Harvard Medical School, Boston, MA, USA;; 2 TB Control Programmes, Geneva, Switzerland;; 3 Quality Health Care Project Tajikistan;; 4 Project Hope;; 5 Republican Centre of Population Protection from Tuberculosis Tajikistan;; 6 Quality Health Care Project Kazakhstan

**Keywords:** external quality assessment, tuberculosis, sputum smeal microscopy, Tajikistan

## Abstract

**Introduction:**

Tajikistan has a laboratory network with three levels of tuberculosis (TB) laboratories. The external quality assessment (EQA) of sputum smear microscopy was implemented in 2007. The objective of this study was to evaluate the EQA system and identify potential performance improvement strategies in TB microscopic laboratories in Sughd, Tajikistan.

**Methods:**

This is a cross-sectional study based on retrospective record review and secondary data analyses on Acid-Fast Bacilli (AFB) microscopy data and EQA reading results collected between the first quarter of 2011 and the fourth quarter of 2013. Descriptive analyses were conducted to examine the overview of microscopy laboratories activities, EQA results, and laboratory performance.

**Result:**

Of the 123,874 smears examined between 2011 and 2013, 11,522 (9.30%) were re-checked by the EQA system. The population TB screening rate rose from 0.46% in 2011 to 0.57% in 2013, and the case positivity rate decreased from 6.98% to 4.80%. The regional EQA results showed a reduction in high false-positive, high false-negative, and low false-negative errors. False-positive errors had decreased from 0.13% in 2011 to 0.07% in 2013, and false-negative errors from 0.91% in 2011 to 0.15% in 2013. Regional sensitivity of smear microscopy, when compared to re-checking controller, increased from 88.2% in 2011 to 97.2% in 2013. The regional specificity level remained relatively stable at above 99%.

**Conclusion:**

Our study found that a decreasing trend of case positivity rate from 2011 to 2013 in Sughd, though the overall laboratory workload was on the rise. In addition, EQA results showed an overall error reduction and an improved sensitivity of smear microscopy in the region. The overview of microscopic laboratory activities and the actual evaluation of the EQA system on sputum smear microscopy complement each other in providing a better picture on the progress of TB laboratory strengthening. We recommend similar approaches to be adapted by future evaluations on TB microscopic laboratories, particularly among countries of high burden. Interactive training and feedback loops are crucial to improving TB surveillance in Tajikistan.

Tuberculosis (TB), an air-borne disease caused by *Mycobacterium tuberculosis,* is the second leading cause of death from an infectious agent in the world.^1^ In 2013, there was an estimated nine million new TB cases globally, and the burden of the disease has hit the low- and middle-income countries most heavily.^1^ Early and accurate TB detection is crucial to effective TB control and care.^2^ Despite the substantial advancement in TB diagnostic and monitoring tools in the past two decades, sputum smear microscopy remains the most common and economical method in the most affected countries.^1^,^2^ Classical smear microscopy, an antiquated test of 130 years, directly identifies Acid-Fast Bacilli (AFB) in Ziehl-Neelsen (ZN) stained sputum under a light microscope.^3^ Simple and inexpensive to adopt, microscopy of sputum smears is particularly suitable for peripheral laboratories based at primary health clinics or district hospitals to quickly detect infectious pulmonary TB cases.^3^ Patients suspected of having pulmonary TB are recommended to have at least two sputum specimens for microscopic examination.^4^ As a result, strengthening laboratory capacity and network to promote good-quality microscopy-based case detection and management has been gaining high priority in the global TB agenda.^5^ The need to establish comprehensive laboratory external quality assessment (EQA) programs under the National TB Programs (NTP) in order to evaluate laboratory performance on AFB microscopy were fully recognized by the global health community. The International Union Against Tuberculosis and Lung Disease (IUATLD) published the guidebook of EQA for AFB microscopy in 2002.^3^ Yet, little research on the evaluation of EQA systems in TB microscopic laboratories has been conducted in resource-limited countries.^5^

Tajikistan is a landlocked Central Asian country that used to be a member of the former Soviet Union. The collapse of the Soviet Union and a half-decade civil war (1992–1998) shortly after the independence of the country heavily damaged both its economy and health infrastructure,^6^,^7^ giving rise to a re-emergence of a TB epidemic that peaked around 2001.^8^ In a population of 8.2 million people, Tajikistan had an estimated prevalence of 12,000 TB cases in 2013.^8^ The country is also one of the 27 high burden countries of multiple drug-resistant TB (MDR-TB); 1 approximately 13% of new patients and 56% of the retreatment TB patients were reportedly MDR-TB cases.^8^ Tajikistan has a well-organized government laboratory network since its formative years (refer to Supplement Figure S1).^5^ Three levels of TB laboratories operate under the umbrella of the Republican TB Center (RTBC) at the central level.^5^ Provincial TB centers form the intermediate level of the laboratory network in the three administrative “oblasts” of the republic: Sughd, Khalton, and Gorno-Badakhsan Autonomous Oblast (GBAO).^5^ Districts of Republican Subordination, a fourth oblast in Tajikistan is centrally managed by the RTBC. The microscopic laboratories are on the peripheral level at the city/district TB dispensaries and primary healthcare clinics. The Sughd Oblast (Sughd) is located in the northwest of Tajikistan with a population of 2.2 million people in 2012.^9^ Sughd has the largest network of TB laboratories in the country with one oblast-level and 22 peripheral laboratories. The oblast first launched the EQA system for AFB microscopy in March 2004 with a grant awarded by the Global Fund. Although outputs of the EQA strengthening work had been consistently monitored and evaluated in funded projects, no operational research was conducted to investigate the actual performance of the EQA system. Our study aimed to evaluate the EQA system and identify potential performance improvements strategies in TB microscopic laboratories in Sughd, Tajikistan.

## Methods

### Description of EQA strategy

In Tajikistan, the government TB laboratories follow the WHO guidelines and grading system of microscopic diagnosis for all AFB smear microscopy readings (refer to Supplement Table S1 and Table S2).^10^ As of 2014, directly observed treatment short course (DOTS) program operates in all 84 microscopic laboratories in Tajikistan. Appropriate quality control procedures are in place in all three levels of TB laboratory service. The staff had been trained in conventional and advanced methods of TB diagnosis and had successfully completed two rounds of EQA provided by Project HOPE. Currently, the RTBC is responsible for coordinating the AFB microscopy network. Quality assurance of smear microscopy services has been implemented with donor assistance since 2007. The collected EQA results are analyzed annually and reported to the NTP management where corrective measures for quality improvement are planned.

**Figure 1:**
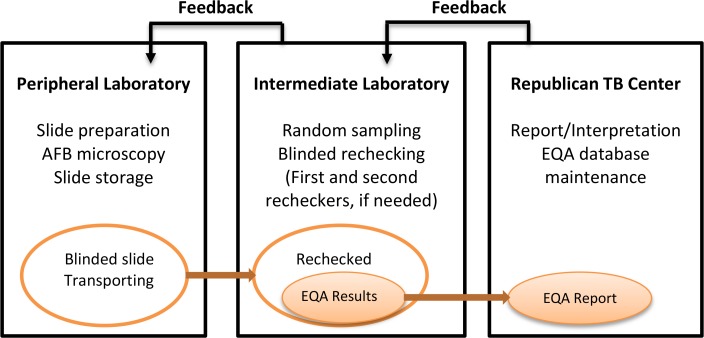
**Algorithm of stepwise rechecking of the EQA system in Sughd^10^**

Figure 1 shows the algorithm of smear evaluation and blinded re-checking of EQA system in the TB microscopic laboratories in Sughd. Tajikistan adopted the lot quality assurance system (LQAS) for its slide sampling strategy with a pre-specified relative sensitivity of 80% and zero acceptance number of errors.

Each year, the National Coordinator at RTBC calculates the needed sampling size for each laboratory based on reported slide positivity rate from the previous year. Oblast and peripheral laboratory staff are notified of the needed number of slides to be submitted for blinded re-checking for each laboratory. Peripheral laboratory staff are responsible of the initial slide preparation, proper slide storage, and physical delivery of all collected slides to the Oblast Laboratory Coordinator during the quarterly re-training sessions. In Sughd, the Oblast Laboratory Coordinator then conducts blinded, random sampling of the AFB smears. The selected AFB smears are blindedly re-examined by the first re-checker at the oblast laboratory using the same technique and number of fields as used in the peripheral laboratories. Slides yielding discrepant results between the peripheral and oblast laboratories are blindedly re-read by a different re-checker at the oblast laboratory or at RTBC, if necessary. The implementation of blinded re-checking is monitored closely by the National Coordinator. The final reading result is reached by the two agreed readings out of the three blinded readings. EQA reading results are forwarded to the National Coordinator for data compilation, entry into the EQA database, and reporting. The National Coordinator conducts quarterly oblast visits to provide feedback to the oblast and peripheral laboratories as well as the original technicians. Table 1 lists the classification of reading errors as defined by the EQA system.^3^

### Study design

We conducted a cross-sectional study on the EQA system of AFB smear microscopy among the government TB laboratories in Sughd, Tajikistan based on retrospective record review and secondary data analyses. Due to resource constraints, the evaluation was not expanded to the national scale. This study did not require ethics review as no human subjects were involved. Quarterly AFB smear microscopy data and the corresponding EQA reading results, collected from the 26 TB laboratories in Sughd (two were in operation for shorter than one year), between the first quarter of 2011 and the fourth quarter of 2013 were extracted from the NRL microscopic laboratory and AFB smear microscopy EQA databases. Database entries were verified against the paper-based EQA reports submitted by the laboratories. Entries missing source documentation were excluded from the analyses. The population served by each laboratory was estimated by the residential population of each corresponding district/municipality as reported by the Agency of Statistics under President of the Republic of Tajikistan.^9^ For districts/municipalities that had more than one peripheral laboratory, annual service population was estimated by dividing the total district/municipal population by the number of active laboratories in each specific year period.

### Data analyses

Descriptive analyses, including t-based confidence interval computation, were performed to examine the overview of microscopic laboratories activities in Sughd in terms of case positivity rate and laboratory workload. Both regional and laboratory-specific measures were calculated. The oblast laboratory (No. 1), due to its distinctive role in the EQA system, and the two peripheral laboratories (No. 24 and 25) that operated shorter than one year, due to their lack of yearly trend, were excluded from the laboratory-specific analyses. Nonetheless, we included data from all these laboratories in the regional-level analyses.

The results of the EQA blinded re-checking system were evaluated based on the proportion of high false-positive (HFP), high false-negative (HFN), low false-positive (LFP), low false-negative (LFN), and quantification errors. Sensitivity and specificity were calculated, along with respective t-based confidence intervals, to demonstrate the AFB smear reading performance of the peripheral laboratories relative to the final EQA re-checking (controller) results. Data were analyzed using *Microsoft Excel (Microsoft Office Excel XP) and Stata 12 software (StataCorp LP, College Station, TX).*

## Results

Out of the 264 expected quarterly EQA reports, 11 were missing from nine peripheral laboratories. These 11 missing reports could possibly be archived in other district files by error. Due to the lack of source documentation and verification failure, their associated quarterly data were excluded from our analyses. From the first quarter of 2011 to the fourth quarter of 2013, TB microscopic laboratories in Sughd examined a total of 123,874 smears, among which, 11,522 (9.30%) were re-checked by the EQA system. The proportion of the population screened had increased from 0.46% in 2011 to 0.57% in 2013. Table 2 gives an overview of the regional and laboratory-specific activities during the study period. While the population in Sughd was steadily on the rise, its case positivity rate had gradually decreased from 6.98% (95% CI: 6.50–7.49%) in 2011 to 4.80% (95% CI: 4.44–5.17%) in 2013, possibly signifying the reduced incidence or prevalence of TB as reflected in WHO global reports. Nonetheless, the overall laboratory workload, reflected by the number of smears examined, grew by 19.5%. On the laboratory level, No. 2, 16 and 23 showed the highest case positivity rates in Sughd while No. 10, 8 and 5 had the heaviest workloads.

Annual EQA rechecking results, as shown in Table 3, reported regional reduction in HFP, HFN and LFN errors. The lowest number of errors was achieved in 2012. Overall, the percentage of FP errors had decreased from 0.13 in 2011 to 0.07 in 2013, though zero FP error was achieved in 2012. The region also saw a reduction of FN errors from 0.91% in 2011 to 0.15% in 2013. On the individual laboratory level, only ten laboratories (45.5%) achieved the NRL’s zero-error standard in 2011. This measure was improved with eighteen laboratories (81.8%) in 2012 and sixteen laboratories (72.7%) in 2013 achieving zero-error. No.18 had the highest number of errors in 2013 (one HFN, two LFP and one QE). Only No. 3 and 7 displayed small rising trends of errors (from zero to one and from zero to two, respectively). No. 11, 10, 13 and 20 showed the most improvement in error elimination.

The performance of TB smear microscopy in Sughd is shown in Table 4. Based on the EQA rechecking results, we reported an increased regional sensitivity from 88.2% (95% CI: 83.4–92.0%) in 2011 to 97.2% (95% CI: 94.3–98.9%) in 2013 (highest at 97.4% (95% CI: 94.7–98.9%) in 2012). The regional specificity level remained relatively stable: 99.9% (95% CI: 99.6–100%) in 2011, 100% (95% CI: 99.9–100%) in 2012 and 99.9% (95% CI: 99.8–100%) in 2013. The positive predictive values and negative predictive values remained stable through the three years (Table 4). Among all low performers in sensitivity were No. 7 (71.4%), 14 (80%), 3 (85.7%), and 21 (90%). They all displayed deteriorating trends in sensitivity over the three years. No. 23 (98.9%) and 18 (99.0%) were the only two laboratories which scored less than perfect in specificity in 2013, though both remained high.

## Discussion

In their review on the roles of laboratories and laboratory systems in effective TB programs, Ridderhof et al., called for more operational research to be done in TB laboratories in the field in resource-limited settings to support evidence-based laboratory practice.^5^ Evaluations of EQA systems and/or blinded random re-checking strategies in TB microscopic laboratories had been conducted in various countries with high TB burden.^6^–^8^ Our study performed the first evaluation on the EQA system and the performance of the TB microscopic laboratories in Sughd, Tajikistan since system implementation.

Our study found a descending trend of case positivity rates from 2011 to 2013 in Sughd, which suggested a regional decline of TB prevalence, while the proportion of population being screened for active TB expanded. This finding is consistent with the stable, national trend of decline in TB prevalence and incidence since 2002^8^, as published by WHO. This could possibly be attributed to higher community awareness of TB and more proactive contact tracing strategies over the past decade. Although earlier a Knowledge, Attitude and Practice (KAP) survey conducted jointly by Project HOPE, WHO Tajikistan, and Sino Project/Swiss Center for International Health in 2005 and 2008 indicated room for improvement in raising public awareness of TB, the two surveys showed improved knowledge of TB symptoms among respondents over a 3-year period.^6^ A qualitative study in 2006, consisting of 13 focus group discussions among 43 community members, echoed that overall knowledge of TB symptoms was accurate among community members.^11^ Since then, the country has implemented various initiatives of patient support groups and community leaders training in expansive scales. Such joint efforts of the NTP and non-governmental organizations might contribute to the climbing case notification rate from 2004 to 2010.^8^ However, since 2010, Tajikistan started to see declining trends in both case notification and incidence rates.^8^ As systematic screening for active TB has been gaining momentum in Central Asia, we anticipate the rising TB screening rate, accompanied by the steadily declining TB incidence and prevalence, to persist with extended local effort in advocacy, communication, and social mobilization activities.

Our study also showed that sputum smear microscopic laboratories in Sughd had achieved total error reduction over these three years, in spite of mounting laboratory workload. Effective reduction in microscopist workload was named the top priority in improving reading quality in previous studies.^7^,^8^ Although our study did not include direct measures of laboratory workforce, we noted that high staff turn-over and emigration of skilled workers continued to challenge local programs in maintaining human resource capacity. As a significant increase in microscopist workforce in the region remained unlikely during the study period, the observed error reduction might be an outcome of stronger interactive training and feedback loop established as part of the EQA system in Sughd. Overall, the peripheral laboratories in this region saw substantial improvement in the sensitivity of AFB smears in TB detection while maintaining high specificity levels. HFN was the most frequent error type found in peripheral laboratories in Sughd, followed by LFP being the second most common error type.

One limitation of our study was that many pieces of EQA data entries in the NRL database were excluded due to missing source documents. Coordinators at RTBC confirmed that all entries were made based on the paper-based reports; however, misfiling of 11 original quarterly reports was possible. Corrective actions that aim to enhance the local filing system for the EQA reports will not only promote data accuracy but also allow future evaluations on other Tajik regions and/or time periods to be completed more efficiently.

With WHO’s recent recommendations on systematic screening for active TB,^12^ continuous monitoring, through laboratory data, on the population TB screening rate, case positivity rate, and number of smears performed gives crucial information on the progress of systematic screening, the trend of TB prevalence, and the status of laboratory workload both at the individual laboratory level and the regional level. Our study computed these three indicators along with the EQA errors and AFB microscopy performance analyses. We believe that the overview of microscopic laboratory activities and the actual evaluation of the EQA system on sputum smear microscopy complement each other in providing a better picture on the progress of TB laboratory strengthening. We recommend similar approaches to be adapted by future evaluations on TB microscopic laboratories, particularly among countries of high burden.

## Supplement

Figure S1: The Structure of Government TB Microscopic Laboratory Network in Tajikistan^13^

**Table S1:** AFB Smear Microscopy Grading system

**Findings**	**Grade**
No acid-fast bacilli found in at least 100 fields	Negative
1–9 acid-fast bacilli per 100 fields	Scanty (report exact figure/100)
10–99 acid-fast bacilli per 100 fields	1+
1–10 acid-fast bacilli per field in at least 50 fields	2+
More than 10 acid-fast bacilli per field in at least 20 fields	3+

**Table S2:** Laboratory numbers and corresponding laboratory names

Laboratory Number	Laboratory Name
1	Deqmoy
2	Khujand G
3	Khujand PHC 1
4	Khujand PHC 5
5	Mastchoq
6	Konibodom PHC
7	Konibodom Sub
8	Isfara
9	Istaravshan
10	Panchakent
11	Kairokkum
12	Chkalovsk
13	Asht
14	Gonchi
15	Zafarobod
16	Mastchoqi Kuqi
17	Spitamen
18	Rasulob
19	Shaqriston
20	Gafurov PHC
21	Gafurov
22	Aini
23	Taboshar
24	Isfara Chorku
25	Gafurov Eva

## Figures and Tables

**Table 1:** Classification of errors^3^

Result of technician	Result of Controllers				

	Negative	1–9 AFB/100f	1+	2+	3+
Negative	Correct	LFN	HFN	HFN	HFN
1–9 AFB/100f	LFP	Correct	Correct	QE	QE
1+	HFP	Correct	Correct	Correct	QE
2+	HFP	QE	Correct	Correct	Correct
3+	HFP	QE	QE	Correct	Correct

* *Note*. LFP = low false-positive, HFP = high false-positive, LFN = low false-negative, HFN = high false-negative, QE = quantification error

**Table 2:** Overview of the annual service population, case positivity rate, total number of smears, and total number of slide rechecking from 2011 to 2013 among TB microscopic laboratories in Sughd, by laboratory

**Laboratory Number**	**Service population**			**Case positivity rate (%)**			**Number of smears examined**			**Number of slides rechecked**		

	**2011**	**2012**	**2013**	**2011**	**2012**	**2013**	**2011**	**2012**	**2013**	**2011**	**2012**	**2013**
**2**	54333	55100	55877	15.03	12.78	13.62	2226	2232	873	58	112	25
**3**	54333	55100	55877	1.60	3.73	2.30	1683	1838	1782	201	73	150
**4**	54333	55100	55877	1.43	3.17	1.45	727	686	1129	90	92	166
**5**	102000	104600	107266	6.35	5.94	6.40	4211	3602	3859	145	201	165
**6**	135400	138000	140650	5.41	4.39	5.01	2926	2280	3473	141	201	208
**7**	46500	47300	48114	0.87	1.33	1.10	691	1266	1659	104	304	309
**8**	232200	236900	120848	4.88	3.47	2.82	4277	3926	4602	141	284	248
**9**	224100	229700	235440	8.99	9.97	4.45	1104	1266	1782	96	118	73
**10**	251000	257900	264990	8.58	5.62	5.50	3812	4503	5273	102	78	105
**11**	40600	41500	42420	7.14	3.57	2.29	490	480	635	88	132	191
**12**	28400	29400	30432	3.68	4.85	5.37	498	588	1106	129	129	293
**13**	136300	140000	143800	9.27	7.67	5.18	510	888	633	155	123	140
**14**	139500	143300	147204	3.73	2.33	0.30	1073	1572	3135	170	225	172
**15**	60800	62500	64248	2.83	6.79	1.97	1452	1113	964	95	252	130
**16**	21100	21600	22112	17.79	3.13	8.33	552	128	52	40	81	28
**17**	116600	119400	122267	5.30	3.73	2.25	2648	2664	3507	209	193	198
**18**	114000	116700	119464	2.71	2.33	2.90	2373	2081	2514	206	156	213
**19**	34900	35800	36723	2.82	1.26	1.63	739	1051	818	135	239	277
**20**	159050	162550	110751	4.47	2.14	2.23	1691	2815	2502	365	298	319
**21**	159050	162550	110751	3.50	0.47	4.35	680	724	561	244	306	444
**22**	72000	73100	74217	4.90	6.06	5.22	394	394	500	83	162	283
**23**	14400	14700	15006	17.97	12.14	13.71	572	650	693	44	76	99

**Regional (95% CI)**	**2251700**	**2302700**	**2354855**	**6.98 (6.50–7.49)**	**5.64 (5.22–6.09)**	**4.80 (4.44–5.17)**	**38437**	**39452**	**45985**	**3063**	**3866**	**4593**

**Table 3:** Annual EQA rechecking results, by laboratory

**Laboratory Number**	**Type of errors**														

	**HFP**			**HFN**			**LFP**			**LFN**			**QE**		
				
	**2011**	**2012**	**2013**	**2011**	**2012**	**2013**	**2011**	**2012**	**2013**	**2011**	**2012**	**2013**	**2011**	**2012**	**2013**
**2**	0	0	0	0	0	0	0	0	0	0	0	0	0	0	0
**3**	0	0	0	0	0	1	0	0	0	0	0	0	0	0	0
**4**	0	0	0	0	0	0	0	0	0	0	0	0	0	0	0
**5**	1	0	0	1	0	0	0	0	0	0	1	0	0	0	0
**6**	0	0	0	2	0	0	0	0	0	0	0	0	0	0	0
**7**	0	0	0	0	0	2	0	0	0	0	0	0	0	0	0
**8**	0	0	0	0	0	0	0	0	0	0	0	0	0	0	0
**9**	0	0	0	1	0	0	0	0	0	0	0	0	0	0	0
**10**	0	0	0	3	0	0	0	0	0	0	0	0	0	0	0
**11**	0	0	0	5	1	0	0	0	0	0	0	0	1	0	0
**12**	0	0	0	0	0	0	0	0	0	0	0	0	0	0	0
**13**	0	0	0	0	0	0	0	0	0	3	0	0	0	0	0
**14**	1	0	0	1	0	1	0	0	0	0	0	0	0	0	0
**15**	0	0	0	0	0	0	0	0	0	0	0	0	0	0	0
**16**	0	0	0	0	0	0	0	0	0	0	0	0	0	0	0
**17**	0	0	0	0	0	0	0	0	0	0	0	0	0	0	0
**18**	0	0	0	3	0	1	0	0	2	2	0	0	0	0	1
**19**	0	0	0	0	0	0	0	0	0	0	0	0	0	0	0
**20**	0	0	0	3	0	0	0	0	0	0	0	0	0	0	0
**21**	0	0	0	0	2	0	1	0	0	0	0	1	0	0	0
**22**	0	0	0	2	3	0	0	0	0	0	0	0	0	0	0
**23**	0	0	0	0	0	0	1	0	1	0	0	0	0	0	0

**Regional**	**2**	**0**	**0**	**21**	**6**	**6**	**2**	**0**	**3**	**7**	**1**	**1**	**1**	**0**	**1**

**Table 4:** Percentage of sensitivity and specificity of smear microscopy, by laboratory

**Laboratory Number**	**Sensitivity (%)**			**Specificity (%)**			**Positive Predictive Value (%)**			**Negative Predictive Value (%)**		

	**2011**	**2012**	**2013**	**2011**	**2012**	**2013**	**2011**	**2012**	**2013**	**2011**	**2012**	**2013**
**2**	100	100	100	100	100	100	100	100	100	100	100	100
**3**	100	100	85.7	100	100	100	100	100	100	100	100	9903
**4**	100	100	100	100	100	100	100	100	100	100	100	100
**5**	94.4	96.2	100	99.2	100	100	94.4	100	100	99.2	99.3	100
**6**	89.5	100	100	100	100	100	100	100	100	98.4	100	100
**7**	NA	100	71.4	100	100	100	NA	100	100	100	100	99.3
**8**	100	100	100	100	100	100	100	100	100	100	100	100
**9**	96.2	100	100	100	100	100	100	100	100	98.6	100	100
**10**	78.6	100	100	100	100	100	100	100	100	96.7	100	100
**11**	58.3	88.9	100	100	100	100	100	100	100	93.8	99.2	100
**12**	100	100	100	100	100	100	100	100	100	100	100	100
**13**	80.0	100	100	100	100	100	100	100	100	97.9	100	100
**14**	87.5	100	80.0	99.4	100	100	87.5	100	100	99.4	100	99.4
**15**	100	100	100	100	100	100	100	100	100	100	100	100
**16**	NA	100	NA	100	100	100	NA	100	NA	100	100	100
**17**	100	100	100	100	100	100	100	100	100	100	100	100
**18**	58.3	100	92.3	100	100	99.0	100	100	85.7	97.5	100	99.5
**19**	100	100	100	100	100	100	100	100	100	100	100	100
**20**	89.7	100	100	100	100	100	100	100	100	99.1	100	100
**21**	100	66.7	90.0	99.6	100	100	85.7	100	100	100	99.3	99.8
**22**	50.0	82.4	100	100	100	100	100	100	100	97.5	98.0	100
**23**	100	100	100	97.4	100	98.9	83.3	100	85.7	100	100	100

**Regional (95% CI)**	**88.2 (83.4–92.0)**	**97.4 (94.7–98.9)**	**97.2 (94.3–98.9)**	**99.9 (99.6–100)**	**100 (99.9–100)**	**99.9 (99.8–100)**	**98.1 (95.3–99.5)**	**100 (98.6–100)**	**98.8 (96.5–99.7)**	**99.0 (98.6–99.3)**	**99.8 (99.6–99.9)**	**99.8 (99.7–99.9)**
