# Supplementary figures and images for: External Quality Assessment of Sputum Smear Microscopy in Tuberculosis Laboratories in Sughd, Tajikistan
Source: Cent Asian J Glob Health. 2016 Mar 4;4(2):230. doi: 10.5195/cajgh.2015.230 (PMC5661208; doi:10.5195/cajgh.2015.230)

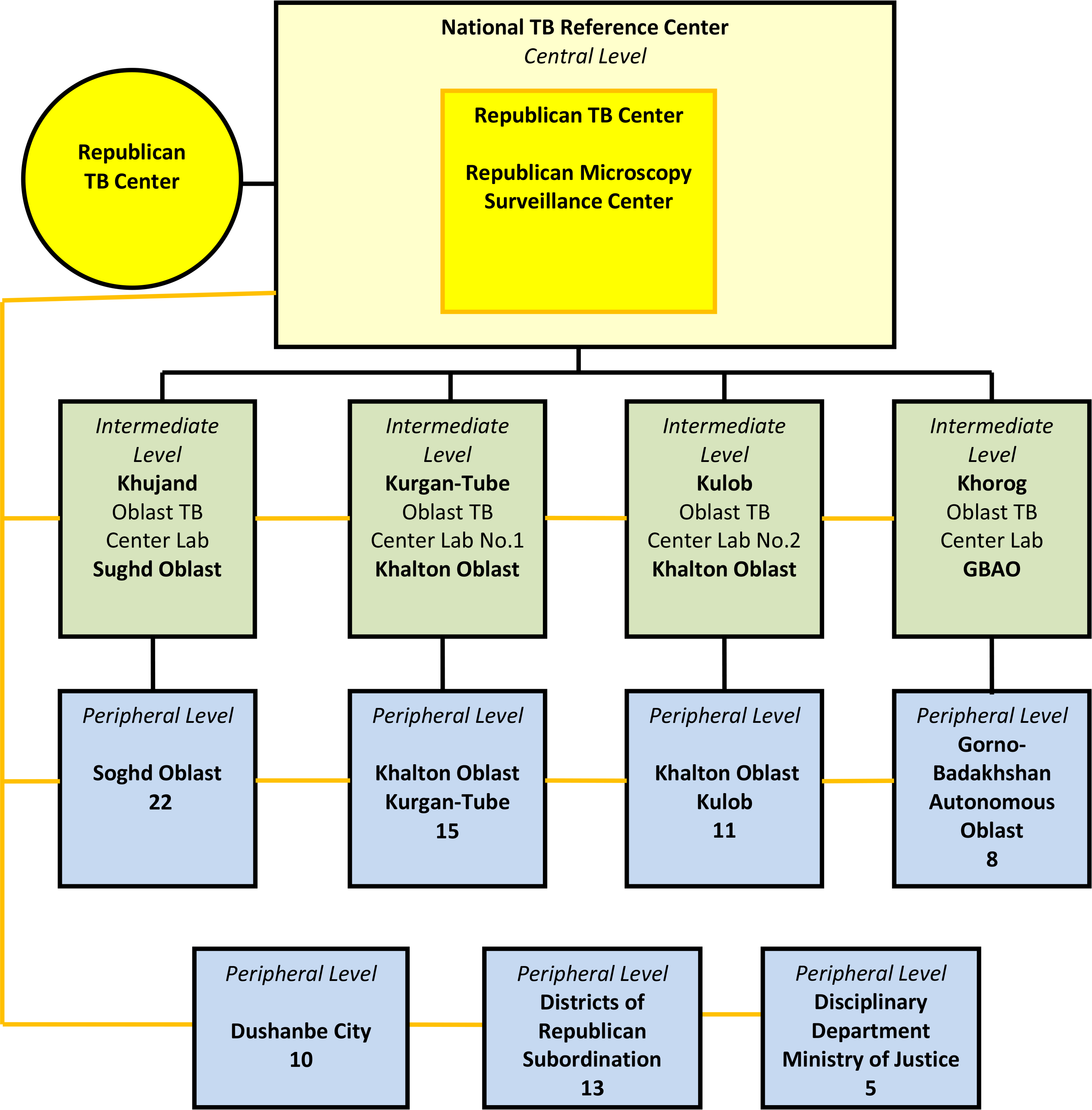

Supplement: Figure S1: — The Structure of Government TB Microscopic Laboratory Network in Tajikistan13 [file cajgh-04-230f2.tif]
